# Characterization of Volatile Profiles and Marker Substances by HS-SPME/GC-MS during the Concentration of Coconut Jam

**DOI:** 10.3390/foods9030347

**Published:** 2020-03-17

**Authors:** Hao Zhang, Haiming Chen, Wenzhu Wang, Wenxiao Jiao, Wenxue Chen, Qiuping Zhong, Yong-Huan Yun, Weijun Chen

**Affiliations:** 1 College of Food Sciences & Engineering, Hainan University, 58 People Road, Haikou 570228, China; zh17733102014@163.com (H.Z.); hmchen168@126.com (H.C.); wwz15606702855@163.com (W.W.); jwx709140172@163.com (W.J.); hnchwx@163.com (W.C.); hainufood88@163.com (Q.Z.); yunyonghuan@foxmail.com (Y.-H.Y.); 2 Chunguang Agro-Product Processing Institute, Wenchang 571333, China

**Keywords:** coconut jam, volatile profiles, HS-SPME/GC-MS, marker substances, PCA

## Abstract

Characteristic aromas are usually key labels for food products. In this study, the volatile profiles and marker substances of coconut jam during concentration were characterized via sensory evaluation combined with headspace solid phase microextraction-gas chromatography-tandem mass spectrometry (HSPME/GC-MS). A total of 33 aroma compounds were detected by HSPME/GC-MS. Principal component analysis revealed the concentration process of coconut jam can be divided into three stages. In the first stage, esters and alcohols were the two main contributors to the aroma of the coconut jam. Next, a caramel smell was gradually formed during the second stage, which was mainly derived from aldehydes, ketones and alcohols. The concentration of aldehydes increased gradually at this stage, which may be the result of a combination of the Maillard reaction and the caramelization reaction. In the final sterilization stage, the ‘odor intensity’ of caramel reached the maximum level and a variety of aroma compounds were produced, thereby forming a unique flavor for the coconut jam. Finally, furfural fit a logistic model with a regression coefficient (*r*^2^) of 0.97034. Therefore, furfural can be used as a marker substance for monitoring the concentration of coconut jam.

## 1. Introduction

The production of jam is one of the oldest food preservation techniques that allows people to enjoy all kinds of fruits during the off-season. According to European Union standards, jams, honey and dried fruits are classified as high-sugar, low-water products [1]. In China, coconut is mainly distributed in the Wenchang area along the coast of the Hainan province. Coconut water and pulp contain a variety of nutrients and have a unique flavor. Coconut water is considered a healthy beverage because it contains a variety of vitamins and minerals, enzymes with anti-inflammatory properties, and antioxidants [2]. In mature coconuts, coconut pulp is commonly used in coconut milk production [3,4]. The physical and chemical properties of coconut pulp make this food suitable for consumption in natural conditions. In addition, coconut pulp is an excellent raw material for the jam industry. However, little research has been conducted on coconut jam. Product development and formulation research are essential parts of the jam industry [5]. The quality of jam is normally defined according to its flavor, color and texture. Among these fundamental properties, aroma as the sensory indicator plays a key role in evaluating the quality of products. From an industrial point of view, an efficient procedure is required to control the quality and stability of the products. Aroma may be a good option to monitor production as a marker [6]. Generally, heating to a high temperature can improve the variety of aroma compounds in coconut jam products and create a unique flavor. Aroma compounds have been identified in coconut water, including alcohols, aldehydes, esters, and acids [7]. Moreover, the flavor perceived by consumers is mainly due to many volatile compounds at different concentrations. Therefore, it is particularly important to explore the changes of the characteristic aroma compounds in the coconut jam during processing.

Due to the advantages of solvent free sample processing, high sensitivity and reliability, headspace solid phase microextraction (HS-SPME) combined with gas chromatography-mass spectrometry (GC-MS) has been widely used for the analysis of volatile compounds [8,9,10,11,12]. The purpose of this study was to verify the volatile compounds of coconut jam produced during the concentration process. Through statistical analysis of a number of aroma compounds, the characteristic aroma compounds appearing in each concentration stage were determined. The marker aroma substances were combined with the concentration time to fit the kinetic equation, which may achieve the monitor production of coconut jam in the industry.

## 2. Materials and Methods

### 2.1. Materials

The fresh coconut pulp was procured from Hainan Taifengyuan Industrial Co., Ltd. (Haikou, China). The sugar was purchased from a local supermarket, while the pectin (degree of esterification<50%) and modified starch were purchased from Guangzhou Dilian Trading Co. Ltd. (Guangzhou, China). The other additives (sucrose ester, soy protein isolate, maltodextrin, fructose syrup and carboxymethyl cellulose) were procured from the Guangzhou Xinzhiwei Food Ingredients mall (Guangzhou, China).

The formula (Table 1) preparation method was provided by local food companies. Briefly, the desired amounts of sugar, maltodextrin and additives were added to the coconut pulp and then the mixture was transferred to an open stainless-steel pan. Batch heating was achieved in a convection oven, and the heating times were 0, 4, 8, 12, 16 and 20 min, respectively. The canning sterilization was conducted at 121 °C for 20 min. The prepared samples were placed in aluminum-foil pouches, sealed and stored at −20 °C.

### 2.2. Sensory Analysis of Aroma

The sensory evaluation panel consisted of ten members (aged 20–30) including male and female members of the college of Food Science and Engineering of Hainan University. They were trained in basic odor recognition tests before performing analysis, according to the references [13,14,15] (Table 2). All the evaluations were conducted at the Fruit and Vegetable Processing Laboratory of Hainan University. The coconut jam samples were stored at −20 °C and were taken out 2 h before serving. Since the study focused on the characteristic aromas of coconut jams during the concentration process, the jams were evaluated for odor only. Before the formal odor assessment, the coconut jam packaging was checked to ensure that it was intact and free of air leaks. All the samples were placed in 50 mL cups and evaluated at room temperature. During the evaluation, the samples were sealed with polyethylene film to protect their aroma from volatilization. The samples were evaluated based on a 5-point intensity scale ranging from 1 ‘low’ (barely detectable) to 5 ‘high’ (moderately detectable). The mean values of these sensory properties were evaluated as the ‘odor intensity’.

### 2.3. Headspace Solid Phase Microextraction of Volatiles

The headspace solid phase microextraction (HS-SPME) method was appropriately modified based on the reference of Liu et al. [16]. Coconut jam (1 ± 0.01 g) was injected into a headspace vial (20 mL) with a syringe and incubated in a water bath at 40 °C for 40 min. The volatile components were extracted with SPME fiber coated with divinylbenzene/carboxen/polydimethylsiloxane (DVB/CAR/PDMS, 50/30 µm Supelco, America) [17,18]. Before analysis, the fibers were conditioned and thermally cleaned by inserting them into the injector port of the GC system at 270 °C for 30 min in a stream of helium, and the aromatic compounds were absorbed by the SPME fiber in the headspace vial at 40 °C for 40 min. The desorption of volatile compounds from the SPME fiber in the GC injector was performed for 5 min, and then analyzed using the GC-MS.

### 2.4. Gas Chromatography-Tandem Mass Spectrometer

Gas chromatography-tandem mass spectrometry (GC-MS) method was appropriately modified based on the reference of Yang et al. [19]. The volatile compounds of the coconut jam were analyzed with a GC - MS instrument (GC-MS-QP2010, Shimadzu, Kyoto, Japan). The analysis was performed on a ZB-5MS silica capillary column (30 m × 0.25 mm, 0.25 μm) equipped with a mass detector. Helium (99.999% purity) was used as the carrier gas with a flow rate of 1.0 mL/min. The temperature of both the injector and detector was set at 270 °C. The programmed sequence for the column was set as follows: an initial temperature of 45 °C was held for 4 min and increased at 5 °C/min to 150 °C/min prior to being increased to 220 °C at 10 °C/min and held at 220 °C for 5 min. The mass detector was equipped and set in electron impact mode at an ionization voltage of 70 eV in the 50–500 amu (atomic mass unit) scan range for mass spectrum collection, and the ion source temperature was 250 °C.

Volatile compounds in coconut jam were identified according to the method of Choi et al. [20]. The volatile compounds were identified by searching the NIST spectrometry library and the retention index (RI) was calculated using a linear heating formula (Equation (1)). Finally, the chemical structure with the closest similarity to the mass spectrum and RI value was selected as the best identification result. Quantitative (relative content) analysis of aroma substances was achieved by peak area normalization [3]. The final volatile components and relative contents determined are shown in Table A1.

(1) $$\mathrm{RI}=100_{n}+\frac{100\left(t_{x}-t_{n}\right)}{t_{n+1}-t_{n}}$$

where t_x_, t_n_, and t_n+1_ are the retention times of the outflow peaks of the component analyzed and the n-alkanes (t_n_ < t_x_ < t_n+1_) with carbon numbers of n and n + 1, respectively.

### 2.5. Kinetics of Furfural Formation

Kinetic models can be used to predict the formation of compounds. Knol et al. used a logistic model to predict the formation and degradation of acrylamide in potato chips [21,22]. The formation kinetics of furfural in the coconut jams during the concentration process is consistent with the four-parameter logistic equation:

(2) $$Y=\frac{A_{1}-A_{2}}{1+{\left(\frac{t}{C}\right)}^{K}}+A_{2}$$

where A_1_ is the minimum content of furfural, A_2_ is the maximum content of furfural, t is the concentration time, C is the concentration at the inflection point during the formation of furfural and K is the slope at the inflection point during the formation of furfural.

### 2.6. Statistical Analysis

The data for different aroma compounds were presented as means ± standard errors. Each class of volatile compounds and sensory data were subjected to the analysis of variance (ANOVA), and the significance of the difference between means was determined by Duncan’s multiple range test (*p* < 0.05) using SPSS 21.0 statistical software (SPSS Inc., Chicago, IL, USA). The correlation matrix analysis was performed on the mean of aroma data by using principal component analysis (PCA). The cluster analysis was conducted by R (The University of Auckland, Auckland, New Zealand). The type of linkage method and distance measurement were “complete” and “Euclidean”, respectively. Logarithmic transformation of raw data was required to be processed before constructing a heat map. Kinetic fit analysis used OriginPro8 (Origin Lab Inc., Northampton, Massachusetts, USA) software.

## 3. Results and Discussion

### 3.1. Sensory Analysis

**Figure 1:**
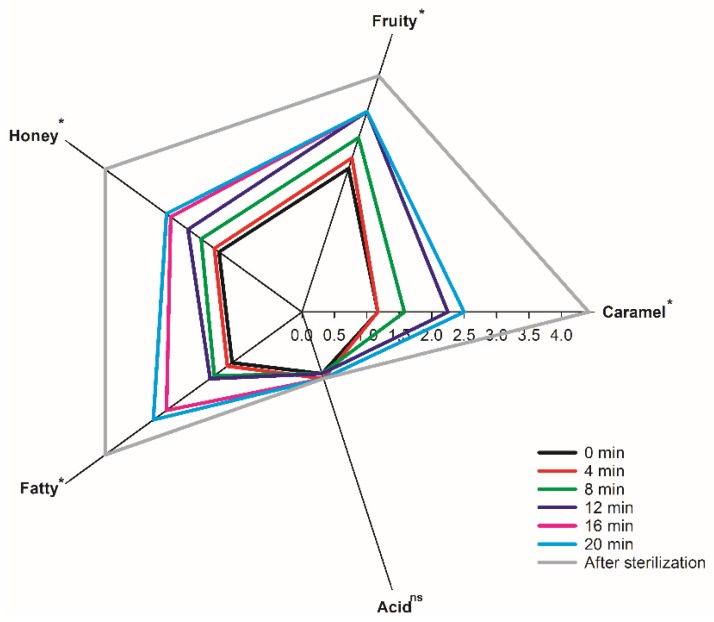
Flavor profiles for the coconut jams (0, 4, 8, 12, 16, 20 min, and after sterilization). The treatments were evaluated in triplicate by 10 panelists (*n* = 30). Asterisks and “ns” indicate significant (* *p* ≤ 0.05) differences and no significant differences of means, respectively.

The aroma sensory evaluation of the coconut jam samples from 0 min to post-sterilization are shown in Figure 1. According to the sensory evaluation radar fingerprint chart, all the samples displayed a combination of fruity, honey, caramel and fatty, but presented almost no acid flavor. As the time of concentration increased, the ‘odor intensity’ of the aroma increased, especially for post-sterilization, which presented higher values significantly than those of other samples (*p* < 0.05). These results showed that the comprehensive aroma of the coconut jams enhanced greatly after the high temperature sterilization. The coconut jam samples from 0 to 8 min seemed to be characterized by more fruity and honey flavors (induced by the higher contents of ester and alcohol in coconut jam) [23]. According to the results of GC-MS as shown in Table A1, the relative contents of ethyl decanoate and 2-octanol were higher than those of other esters and alcohols in the time range of 0–8 min. It is worth noting that ethyl decanoate had a fruity odor and 2-octanol had aromatic characteristics [24,25]. Therefore, the odor intensity of fruit and honey in coconut jam was more obvious during this period. As the heating continued from 8 min to the end of the sterilization, the caramel aroma gradually became the dominant aroma. This may be due to the Maillard and caramelization reaction during high temperature because hexanal, furfural and benzenecarbonal were detected during this period [26,27]. It is worth mentioning that the relative contents of hexanal and furfural began to rise significantly after 8 min, and benzenecarbonal itself has the characteristics of caramel aroma [28,29]. Therefore, it is possible that the combined effect of these three compounds ultimately leads to a significant increase in the caramel odor intensity.

### 3.2. HS-SPME/GC-MS Analysis Results

**Figure 2:**
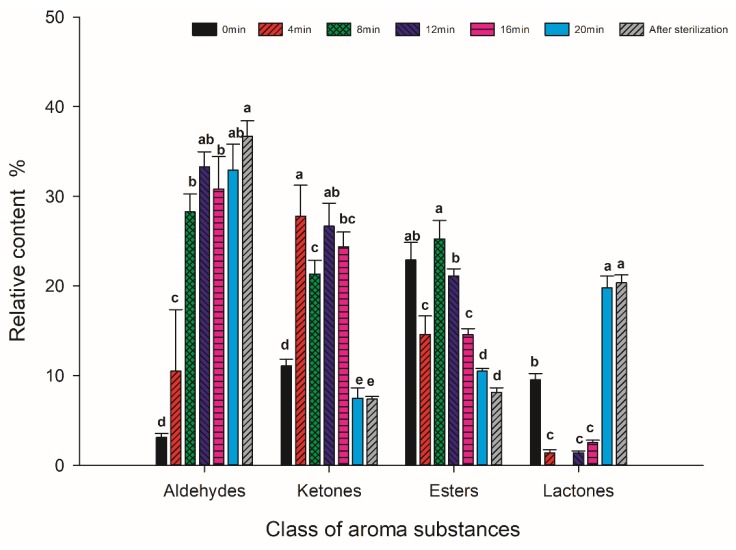
Relative contents of ester, lactone, alcohol and ketone aroma compounds for the coconut jam obtained by GC-MS. Values identified with different letters represent significant differences for each class of compound.

**Figure 3:**
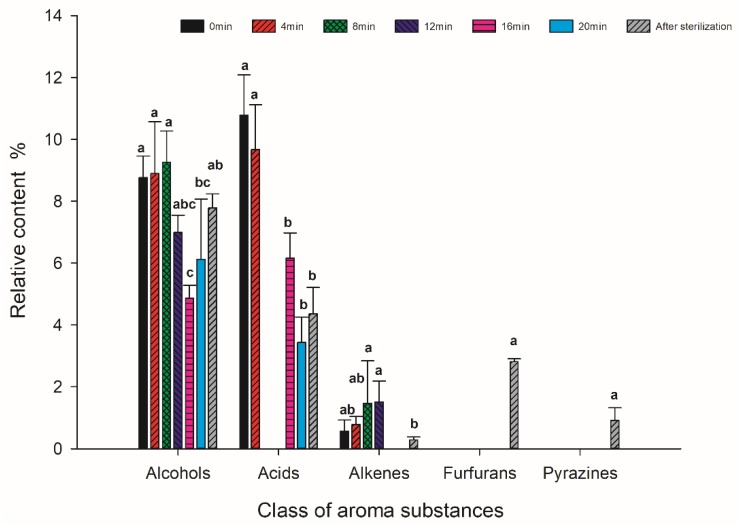
Relative contents of aldehydes, acids, alkenes, furfurans and pyrazines for the coconut jam obtained by GC-MS. Values identified with different letters represent significant differences for each class of compound.

A total of 33 different aroma compounds were detected. According to the relative content of each group of aroma components determined, the components were arranged in order from highest to lowest: aldehydes, ketones, esters, lactones, alcohols, acids, alkenes, furfurans and pyrazines (Figure 2 and Figure 3). The relative content of aldehydes was the highest among the nine types of aroma compounds, accounting for approximately 29.7% of the total flavor components of each coconut jam sample.

#### 3.2.1. Esters and Lactones

Ester compounds play an important role in the aroma of coconut products. Esters are formed by the enzymatic condensation of organic acids and alcohols [30]. In fresh coconut water, the main esters are ethyl hexanoate, ethyl octanoate, ethyl decanoate and ethyl dodecanoate. In particular, ethyl decanoate and ethyl dodecanoate are present at the highest concentrations and represent approximately half of all the esters present [23,31]. Ethyl decanoate, ethyl dodecanoate and hexyl formate represented about 82%–92% of the total ester content of the coconut jam samples. As shown in Table A1, coconut jam has the same ester aroma compounds as fresh coconut water, with only a few differences, indicating that the coconut jam basically retains the original flavor of the coconut.

Lactones are cyclic esters with a fruity aroma. In this study, two lactones (delta-nonalactone and delta-dodecalactone) were detected. Delta-nonalactone has fruit and dairy odor characteristics [32]. Delta-dodecalactone has a sweet and fruity scent [33]. It is worth noting that the content of delta-nonalactone kept rising during the heating process and reached the highest value after sterilization, which enhanced the fruit aroma of coconut jam and made its flavor more unique.

#### 3.2.2. Alcohols

Alcohols are formed either by anabolism or catabolism (Ehrlich pathway) of amino acids [30]. These aroma compounds have both positive and negative effects. There are more alcohols in fresh coconut water than in coconut jam. Cappelletti et al. reported that fresh coconut water contained 13 types of alcohols, of which 3-methyl-1-butanol, hexanol, 2-ethyl-1-hexanol, 1-octanol and 1-decanol were present in high amounts, accounting for 72% of all the alcohols [23]. The GC-MS results are shown in Table A1. Five alcohols present in the coconut jam samples were identified as isopentyl alcohol, 2-methyl-1-butanol, pentyl alcohol, 2-octanol and furfuryl alcohol. The content of 2-octanol was the highest among the alcohols from 0 to 20 min. However, furfuryl alcohol became the dominant aroma compound after sterilization and was present at a significantly higher content than the other alcohols (*p* < 0.05). The compound 2-octanol has aromatic odor characteristics, and furfuryl alcohol has caramel aroma characteristics [34]. The formation of furfuryl alcohol and the interaction of this compound with other the aroma compounds imparted a special flavor to the sterilized coconut jam.

#### 3.2.3. Ketones

In total, seven types of ketones were detected in the coconut jam samples. Table A1 shows that 3-hydroxy-2-butanone, 2-octanone, 2-nonanone and 5-hexyl-4-methyldihydro-2(3H)-furanone were detected from 0 to 20 min. Ethylidene acetone, dihydro-2-methyl-3(2H)-furanone and 3-6-dimethyl-tetrahydropyran-2-one were detected after sterilization. The contents of 2-octanone and 5-hexyl-4-methyldihydro-2(3H)-furanone in the ketones were significantly higher than the contents of the other ketones from 0 to 20 min before sterilization (*p* < 0.05), and 2-octanone has soapy and fruity odor characteristics [28]. After sterilization, dihydro-2-methyl-3(2H)-furanone, with sweet and creamy characteristics, interacted with the other ketones to contribute to the aroma of the coconut jams.

#### 3.2.4. Aldehydes

The aldehydes generated during the concentration process of the coconut jams were important precursors in the formation of aromatic compounds such as higher alcohols and esters [35]. From 0 min to post-sterilization, hexanal and furfural always dominated the aldehyde aroma. Hexanal and furfural are common aroma substances in baked and caramel goods flavors. The appearance of these compounds brought a caramel and baking aroma to the coconut jams, which had a positive effect, but the contents of these compounds should not be too high because this condition would mask the fruit flavor of the coconut jams.

#### 3.2.5. Acids and Alkenes

The analysis results of the GC-MS show that only two types of acids and one alkene were detected in the coconut jam samples. The acids were mainly composed of decanoic acid, and the alkenes were mainly composed of (3E)-6-methyl-3-undecene. In general, the contributions of acids and alkenes to the aroma of the coconut jams were comparatively low, due to the presence of compounds that were not particularly odor active. Decanoic acid was present at a relatively high content in the acids, and has rancid and fatty odor characteristics [28] that may negatively affect the overall aroma of coconut jam. As seen from Table A1, the content of decanoic acid gradually decreased from 0 min to post-sterilization. The acids were formed during the early stages of the concentration process. Therefore, to reduce the adverse effects of volatile acids on the overall aroma, the concentration temperature and the agitation speed should be closely controlled in the early processing of coconut jam.

#### 3.2.6. Furans and Pyrazines

The furan and pyrazine aromatic compounds were detected after sterilization. These compounds may have been the result of a combination of the Maillard reaction and caramelization reaction during high temperature sterilization [36,37]. As shown in Table A1, 2-pentylfuran comprised the largest fraction of the furans, and this compound has a fruity fragrance [38]. Therefore, this compound was an aroma compound that had a positive effect on the flavor of coconut jam post-sterilization.

### 3.3. Changes in the Aroma Components of Coconut Jams during Concentration

**Figure 4:**
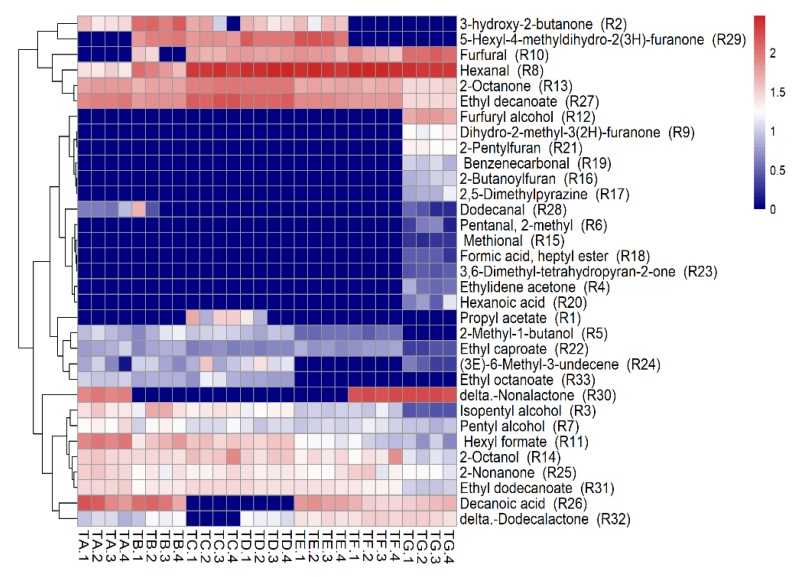
Heat map of the contents of the main volatile compounds during the concentration times and after sterilization. The content value increases with the color varying from blue to red. (TA1…4, TB1…4 to TG1…4) represent samples in quadruplicate at different time points.

The statistical analysis software, R, was used to form cluster heat maps to visually show the differences in the aroma compound contents at different concentration times. As shown in Figure 4, the main aroma compounds displayed various trends. All the aroma compounds in the seven groups of processed coconut jam can be divided into two clusters. R2–R27 (from 3-hydroxy-2-butanone to ethyl decanoate) were grouped into one class, and R12–R32 (from furfuryl alcohol to delta-dodecalactone) were grouped into another class. The amount of furfural and hexanal constantly increased from 0 min to post-sterilization, whereas the amounts of 2-octanone and ethyl decanoate first increased from 0 to 12 min but then gradually decreased. Conversely, the contents of pentyl alcohol, isopentyl alcohol, hexyl formate, ethyl caproate 2-octanol, 2-nonanone and ethyl dodecanoate decreased gradually. These phenomena may be due to the use of sugar as a precursor substance to generate important flavor substances; sugar generates a variety of aroma compounds when heated to a high temperature and produces aroma substances such as furan derivatives and ketone aldehydes [39]. In addition, during the heating process, the original heat unstable aroma substances in the coconut water were degraded, such as alcohols, ketones, and esters [40]. The Maillard reaction also causes changes in flavor, carbonyl compounds forming by oxidation of ketones and aldehydes, and reduction of sugars. Further, carbonyl compounds and amino acids undergo oxidation, decarboxylation, condensation, and cyclization to form a series of reactive intermediates [41]. However, these reactive intermediates continue to react with amino acids, ultimately causing changes in the flavor of the product.

**Figure 5:**
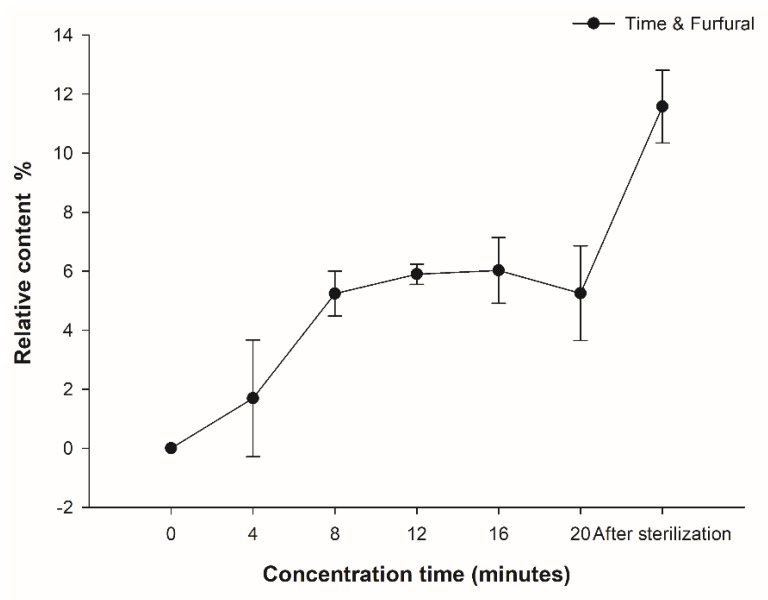
The variation of furfural content during different concentration times.

It was a reasonable and novel choice to use aromatic compounds as production indicators [6,42]. As shown in Figure 5, the relative content of furfural increased with increasing concentration time, and the two turning points in the growth process occurred between 4 and 8 min and between 20 min and post-sterilization, which was consistent with the PCA analysis results. Moreover, the formation kinetics of the furfural in the coconut jam samples during concentration were fitted to a logistic model. We inserted the parameter values in the model equation to ultimately obtain the kinetic equation of the furfural formation (Equation (3)). The logistic model has a high degree of fit (*r*^2^ = 0.97034).

(3) $$Y=\frac{-5.72899}{1+{\left(\frac{t}{4.78625}\right)}^{4.90798}}+5.73277$$

Therefore, furfural can be used as a marker aroma in the production of coconut jam to monitor the degree of concentration of the product.

### 3.4. Principal Component Analysis (PCA) of the Characteristic Aroma of Coconut Jams

Although the quantitative and qualitative analysis could measure the aroma compounds present in the coconut jams at different concentration times, these methods were not able to determine the characteristic aroma components in the samples. PCA is a multivariate statistical analysis method that employs multiple variables to linearly transform the data to select fewer important variables [43]. Thus, this method can be used to determine the characteristic aroma components of the overall aroma of the dominant coconut jam. The first principal component (PC1) and the second principal component (PC2) explain changes in the data variance of 59.35% and 17.84%, respectively. The cumulative contribution rates of PC1 and PC2 reached a high level, which was sufficient to explain the maximum variation of the aroma substances in the coconut jams during the concentration process.

**Figure 6:**
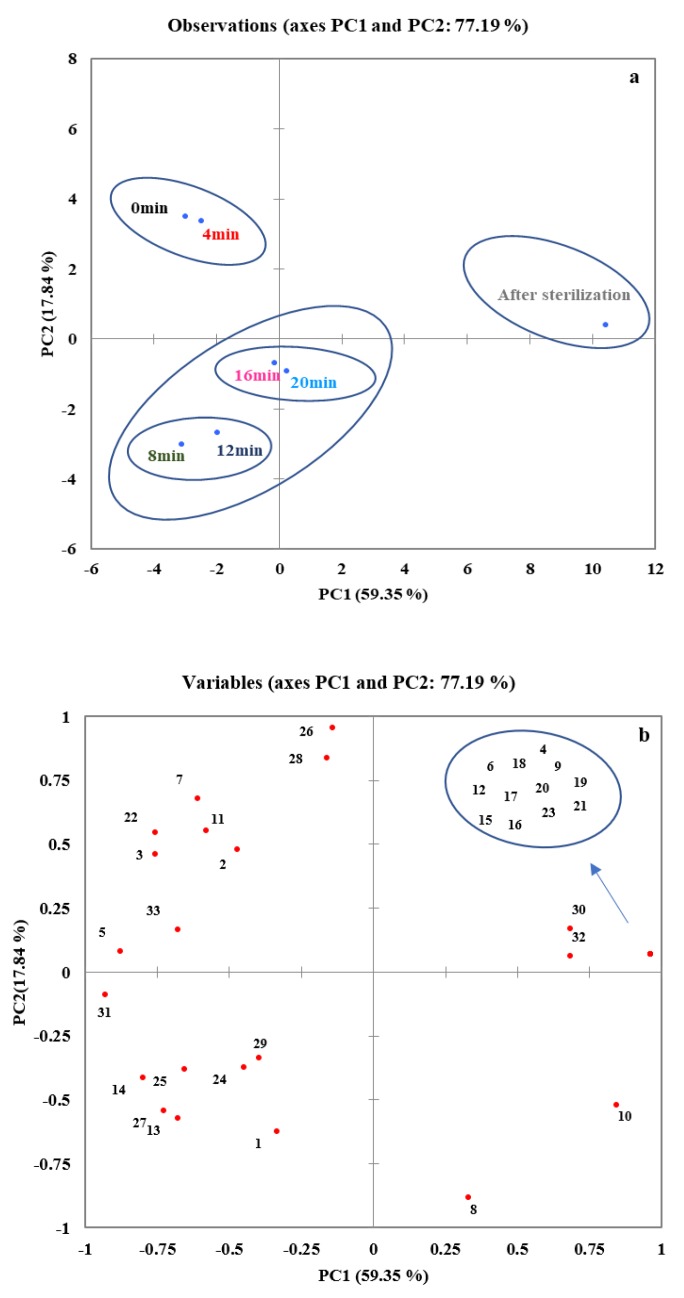
Analysis score (**a**) and correlation loading (**b**) plot of principal components 1 and 2 for the volatile compounds in coconut jam. Coconut jam sample distribution at different concentration times (**a**). Distribution of 33 volatile compounds in coconut samples (**b**).

As shown in the scatterplot of PC1 and PC2 in Figure 6a, seven coconut jam samples were clearly distributed in the three spatial regions of the PCA plot. The after-sterilization samples were located in the region covering the positive axis of PC1 and the positive axis of PC2. The associated aroma components could be ethylidene acetone (4), pentanal-2-methyl (6), dihydro-2-methyl-3(2H)-furanone (9), furfuryl alcohol (12), methional (15), 2-butanoylfuran (16), 2,5-dimethylpyrazine (17), formic acid, heptyl ester (18), benzenecarbonal (19), hexanoic acid (20), 2-pentylfuran (21), 3,6-dimethyl-tetrahydropyran-2-one (23), delta-nonalactone (30) and delta-dodecalactone (32) (Figure 6b); these 14 compounds may be the characteristic aroma components of the post-sterilization samples.

The 0 min and 4 min samples were located in the top left corner of the region covering the negative axis of PC1 and the positive axis of PC2. The aroma associated with the 0 min and 4 min samples could be 3-hydroxy-2-butanone (2), decanoic acid (26), dodecanal (28), ethyl octanoate (33), isopentyl alcohol (3), pentyl alcohol (7), hexyl formate (11), ethyl caproate (22) and 2-methyl-1-butanol (5), implying that these compounds may be the main aroma components of the 0 min and 4 min samples (Figure 6b).

The 8 min, 12 min, 16 min and 20 min samples were mainly located in the bottom left corner of the region covering the negative axis of PC1 and the negative axis of PC2 (20 min was situated along the positive axis of PC1), implying that the aroma was steady from 8 min to 20 min. The aroma associated with these times could be propyl acetate (1), 2-octanone (13), 2-octanol (14), 2-nonanone (25), (3E)-6-methyl-3-undecene (24), ethyl decanoate (27) and 5-hexyl-4-methyldihydro-2(3H)-furanone (29), (Figure 6b), suggesting that these compounds could be the characteristic aroma components at these times. It is worth noting that at this stage it can be divided into two subgroups of 8–12 min and 16–20 min. Among them, the related compounds 2-octanol (14), ethyl decanoate (27), 2-octanone (13) and (3E)-6-methyl-3-undecene (24) decreased significantly from 12–16 min, which may be the continuous rise in temperature leading to the compounds undergoing the process of first synthesis and degradation. Eventually, the flavor of coconut jam changes at this stage.

According to PCA analysis results, the change in the aroma of coconut jam during the concentration process can be divided into three stages: the initial stage, the middle heating stage, and the sterilization stage (Figure 6a). The aroma of coconut jam after high temperature sterilization significantly differed from the aroma of the first two stages, and new flavor compounds such as furans and pyrazines were formed. Therefore, after sterilization, the coconut jam had a relatively obvious caramel aroma. Overall, the results of the PCA show that the odor of coconut jam at each stage was formed by the combined action of multiple individual aroma compounds.

## 4. Conclusions

In conclusion, the volatile profiles and marker substances of coconut jam processing were characterized by HSPME/GC-MS. According to PCA analysis, the concentration process of coconut jam can be regarded as occurring in three stages. The results suggested that esters and alcohols, such as isopentyl alcohol, pentyl alcohol, hexyl formate and ethyl caproate, were the main contributors to the aroma of the coconut jam in the early stage, while 2-octanone, 2-octanol, 2-nonanone and 5-hexyl-4-methyldihydro-2(3H)-furanone were the main aroma components of the middle stage. In the final sterilization stage, a variety of aroma compounds were produced, such as benzenecarbonal, dihydro-2-methyl-3(2H)-furanone and furfuryl alcohol, forming the unique flavor of the coconut jam. The stepwise increase of the furfural content is consistent with the inflection point of the change in aroma during the whole process of coconut jam concentration, and the logistic model has a higher degree of fit, which can be used as a marker of aroma to monitor the concentration of the product. However, the mechanisms of action of certain aroma compounds released from the coconut jam are still unclear. Therefore, it is necessary to find new methods to further study the formation mechanism of aroma compounds in coconut jam.

## Figures and Tables

**Table 1:** Detailed ingredient ratios of coconut jam (proportion of ingredients added per 100 g of coconut jam).

No.	Ingredient	Proportion (%)
1	Coconut pulp	40
2	Fructose syrup	18
3	Sugar	18
4	Maltodextrin	18
5	Modified starch	3.6
6	Soy protein isolate	1.8
7	Carboxymethyl cellulose	0.3
8	Sucrose esters	0.3
9	Monoglyceride	0.3
10	Pectin	0.2

**Table 2:** Description and definition of the aroma of coconut jam.

Categories	Descriptors	Definitions
Odor/Flavor	Fruity	May resemble the odor of coconut, pineapple, apple, or other fruits
	Caramel	Cooked sugar, all which reminds sugar cooking, caramel
	Acid	Sour off-flavor due to acid-producing organisms such as *Lactococcus lactis ssp. cremoris*
	Fatty	Aromatics associated with stale fats
	Honey	Aromatics associated with the sweet fragrance of honey

## Appendix A

**Table A1:** Aroma compounds of coconut jam treated by different concentration time.

No.	Category	RI ^f^	Component Name ^g^	Identification	Area (%)						
					0 min	4 min	8 min	12 min	16 min	20 min	After Sterilization
	Aldehydes										
1		817	Hexanal	MS, RI	2.61 ± 0.49d	7.63 ± 3.10c	23.04 ± 2.55b	27.39 ± 1.47a	24.79 ± 2.67ab	27.67 ± 1.68a	23.48 ± 0.68b
2		1405	Dodecanal	MS, RI	0.52 ± 0.22b	1.6 ± 2.55a	-	-	-	-	0.23 ± 0.07b
3		914	Furfural	MS, RI	-	1.69 ± 1.98c	5.24 ± 0.76b	5.9 ± 0.34b	6.03 ± 1.11b	5.25 ± 1.61b	11.58 ± 1.23a
4		-	Pentanal,2methyl	MS	-	-	-	-	-	-	0.32 ± 0.16
5		1014	Methional	MS, RI	-	-	-	-	-	-	0.18 ± 0.01
6		1190	Benzenecarbonal	MS, RI	-	-	-	-	-	-	0.93 ± 0.18
	Ketones										
1		-	3-hydroxy-2-butanone	MS	2.9 ± 0.75b	11.81 ± 2.18a	2.87 ± 2.74b	3.41 ± 0.94b	3.31 ± 1.22b	-	-
2		998	2-Octanone	MS, RI	6.08 ± 0.32b	5.43 ± 0.54c	9.62 ± 0.81a	9.66 ± 0.13a	5.42 ± 0.23c	4.91 ± 0.34c	3.1 ± 0.17d
3		1281	2-Nonanone	MS, RI	2.12 ± 0.71ab	2.00 ± 0.42ab	2.28 ± 0.34ab	2.25 ± 0.23ab	2.04 ± 0.36ab	2.59 ± 1.16a	1.60 ± 0.26b
4		1480	5-Hexyl-4-methyldihydro-2(3H)-furanone	MS, RI	-	8.57 ± 1.55c	6.6 ± 0.57c	11.35 ± 2.12b	13.64 ± 2.47a	-	-
5		-	Ethylidene acetone	MS	-	-	-	-	-	-	0.62 ± 0.16
6		844	Dihydro-2-methyl-3(2H)-furanone	MS, RI	-	-	-	-	-	-	1.82 ± 0.21
7		1105	3,6-Dimethyl-tetrahydropyran-2-one	MS, RI	-	-	-	-	-	-	0.28 ± 0.02
	Esters										
1		1396	Ethyl decanoate	MS, RI	9.15 ± 0.96c	6.76 ± 0.44d	14.39 ± 1.37a	13.26 ± 0.32b	7.45 ± 0.27d	6.72 ± 0.42d	2.99 ± 0.05e
2		1099	Ethyl caproate	MS, RI	0.75 ± 0.17a	0.66 ± 0.13ab	0.45 ± 0.05c	0.46 ± 0.04c	0.58 ± 0.11bc	0.59 ± 0.07bc	0.24 ± 0.03d
3		1594	Ethyl dodecanoate	MS, RI	3.05 ± 0.25a	2.20 ± 0.2c	2.90 ± 0.12a	2.65 ± 0.11b	2.47 ± 0.11b	2.10 ± 0.08c	0.97 ± 0.09d
4		1183	Ethyl octanoate	MS, RI	0.98 ± 0.11a	0.7 ± 0.04b	0.98 ± 0.42a	0.62 ± 0.03b	-	-	-
5		969	Hexyl formate	MS, RI	9.01 ± 0.01a	4.29 ± 1.79b	3.47 ± 0.45b	3.4 ± 0.39b	1.54 ± 0.22c	1.1 ± 0.31c	0.76 ± 0.29c
6		-	Propyl acetate	MS	-	-	3.08 ± 1.82a	0.78 ± 1.12b	-	-	-
7		1060	Formic acid, heptyl ester	MS, RI	-	-	-	-	-	-	0.27 ± 0.05
	Lactones										
1		1480	delta-Nonalactone	MS, RI	8.58 ± 0.81b	-	-	-	-	16.84 ± 1.38a	17.42 ± 0.80a
2		1692	delta-Dodecalactone	MS, RI	0.97 ± 0.18d	1.41 ± 0.34c	-	1.41 ± 0.2c	2.54 ± 0.27b	2.96 ± 0.23a	2.94 ± 0.34a
	Alcohols										
1		-	Isopentyl alcohol	MS	2.69 ± 0.6ab	3.57 ± 1.80a	2.28 ± 0.22b	1.81 ± 0.24bc	0.99 ± 0.07cd	1.06 ± 0.22cd	0.27 ± 0.02d
2		-	2-Methyl-1-butanol	MS	0.88 ± 0.22a	0.95 ± 0.45a	1.02 ± 0.08a	0.80 ± 0.11a	0.36 ± 0.02b	0.37 ± 0.04b	-
3		-	Pentyl alcohol	MS	2.09 ± 0.46a	1.59 ± 0.39b	1.27 ± 0.21bc	1.11 ± 0.12c	1.04 ± 0.12c	1.24 ± 0.19bc	0.86 ± 0.10c
4		1010	2-Octanol	MS, RI	3.10 ± 0.26abc	2.79 ± 0.16abc	4.7 ± 2.21a	3.28 ± 0.41ab	2.47 ± 0.41bc	3.47 ± 2.44ab	1.17 ± 0.21c
5		947	Furfuryl alcohol	MS, RI	-	-	-	-	-	-	5.49 ± 0.59
	Acids										
1		1373	Decanoic acid	MS, RI	10.78 ± 4.3a	9.67 ± 3.45a	-	-	6.16 ± 0.82b	3.43 ± 0.82b	3.72 ± 0.5b
2		1081	Hexanoic acid	MS, RI	-	-	-	-	-	-	0.64 ± 0.43
	Alkenes										
1		1160	(3E)-6-Methyl-3-undecene	MS, RI	0.57 ± 0.36ab	0.78 ± 0.26ab	1.47 ± 1.37a	1.51 ± 0.67a	-	-	0.29 ± 0.09b
	Furfurans										
1		1015	2-Butanoylfuran	MS, RI	-	-	-	-	-	-	0.91 ± 0.18
2		1090	2-Pentylfuran	MS, RI	-	-	-	-	-	-	1.9 ± 0.11
	Pyrazines										
1		1020	2,5-Dimethylpyrazine	MS, RI	-	-	-	-	-	-	0.92 ± 0.41

a–e Different letters in the same row are significantly different. ^g^ The compounds were identified by comparing retention indices of authentic n-alkanes (C8–C40) and mass spectra with those from NIST08 and NIST08s. ^f^ Retention indices (RIs) were based on a series of alkanes (C8–C40). Labels 0, 4, 8, 12, 16, 20 min and after sterilization represent the different concentration processing times.
